# Comparison of nasotracheal versus orotracheal intubation for sedation, assisted spontaneous breathing, mobilization, and outcome in critically ill patients: an exploratory retrospective analysis

**DOI:** 10.1038/s41598-023-39768-1

**Published:** 2023-08-03

**Authors:** Jörn Grensemann, Sophie Gilmour, Pischtaz Adel Tariparast, Martin Petzoldt, Stefan Kluge

**Affiliations:** 1 Department of Intensive Care Medicine, University Medical Center Hamburg-Eppendorf, Martinistraße 52, 20246 Hamburg, Germany; 2 Department of Anesthesiology, University Medical Center Hamburg-Eppendorf, Martinistraße 52, 20246 Hamburg, Germany

**Keywords:** Outcomes research, Risk factors

## Abstract

Nasotracheal intubation (NTI) may be used for long term ventilation in critically ill patients. Although tracheostomy is often favored, NTI may exhibit potential benefits. Compared to orotracheal intubation (OTI), patients receiving NTI may require less sedation and thus be more alert and with less episodes of depression of respiratory drive. We aimed to study the association of NTI versus OTI with sedation, assisted breathing, mobilization, and outcome in an exploratory analysis. Retrospective data on patients intubated in the intensive care unit (ICU) and ventilated for > 48 h were retrieved from electronic records for up to ten days after intubation. Outcome measures were a Richmond Agitation and Sedation Scale (RASS) of 0 or − 1, sedatives, vasopressors, assisted breathing, mobilization on the ICU mobility scale (ICU-MS), and outcome. From January 2018 to December 2020, 988 patients received OTI and 221 NTI. On day 1–3, a RASS of 0 or − 1 was attained in OTI for 4.0 ± 6.1 h/d versus 9.4 ± 8.4 h/d in NTI, p < 0.001. Propofol, sufentanil, and norepinephrine were required less frequently in NTI and doses were lower. The NTI group showed a higher proportion of spontaneous breathing from day 1 to 7 (day 1–6: p < 0.001, day 7: p = 0.002). ICU-MS scores were higher in the NTI group (d1–d9: p < 0.001, d10: p = 0.012). OTI was an independent predictor for mortality (odds ratio 1.602, 95% confidence interval 1.132–2.268, p = 0.008). No difference in the rate of tracheostomy was found. NTI was associated with less sedation, more spontaneous breathing, and a higher degree of mobilization during physiotherapy. OTI was identified as an independent predictor for mortality. Due to these findings a new prospective evaluation of NTI versus OTI should be conducted to study risks and benefits in current critical care medicine.

## Introduction

In the intensive care unit, endotracheal intubation and consecutive mechanical ventilation are required to treat respiratory insufficiency as well as for different surgical procedures and other interventions^1^. Intubation is commonly performed orotracheally by laryngoscopy after preoxygenation and administration of narcotics and a muscle relaxant. In the continuing course of mechanical ventilation, patients usually require sedation to ensure tolerance of the orotracheal tube but on the other hand, sedation may lead to an increased use of vasopressors, increased rate of delirium, critically ill induced muscular weakness, and may impair spontaneous breathing as well as physiotherapy and patients’ mobilization, and may increase mortality^2,3^. To circumvent these problems and to increase patient comfort, intubation via a nasotracheal approach might be favorable^4,5^. Although this approach has been widely abandoned e.g. due to the potential risk of sinusitis^6,7^, this could not be uniformly linked to nasotracheal intubation and is also of concern with orotracheal intubation^8–10^. In our institution, both routes of intubation are routinely used for tracheal intubation in the intensive care unit, as it has been observed that patients with nasotracheal tubes require less sedation and catecholamines and are more alert. Furthermore, it is claimed that the rate of tracheostomies may be reduced. However, this has not been studied systematically, recently. Therefore, we retrospectively assessed the association between the route of intubation and depth of sedation, vasopressor therapy, physiotherapy, and rate of spontaneous breathing, as well as complications and outcome in an exploratory approach to generate hypotheses for further studies.

## Methods

### Ethics

The retrospective and anonymized data collection and analyses were conducted in accordance with local government law (Hamburg Hospital Act [Hamburgisches Krankenhausgesetz] Sect. 12) without the requirement for approval or informed consent. The study was performed in accordance with the ethical standards as laid down in the 1964 Declaration of Helsinki and its later amendments or comparable ethical standards.

### Study design

This study was a retrospective, single-center, exploratory cohort-study.

### Setting and population

The study was conducted in the Department of Intensive Care, University Medical Center, Hamburg-Eppendorf with twelve intensive care units (surgical, medical, neurological, and interdisciplinary) and a total of 140 beds. Adult patients were included if they had received tracheal intubation in the intensive care unit for clinical indication and had been mechanically ventilated for more than 48 h. Patients were included from January 1, 2018, to December 31, 2020. Patients receiving intubation exclusively for surgery or other procedures were excluded. According to the route of intubation, patients were categorized into either the nasotracheal intubation group (NTI) or the orotracheal intubation group (OTI). Repeated intubations in individual patients were assessed, but only the last episode of intubation and ventilation was included in the analysis of time and outcome variables. For the purpose of patient characterization and adjustment of the analyses, patients were grouped as surgical, neurological, medical with community acquired pneumonia, and other medical patients.

### Outcome parameters

The primary endpoint was the depth of sedation defined as fraction of time with a Richmond-Agitation-Sedation-Scale (RASS) of 0 or − 1 on the first day after intubation to end of day 3. Secondary endpoints were the mean RASS score, doses of vasopressors and sedative medication, proportion of spontaneous breathing, mobilization with physiotherapy assessed by the ICU-mobility scale^11^, rates of successful extubation and tracheostomy, length of ventilation, and complications such as incidence of ventilator acquired pneumonia (VAP), and sinusitis. VAP and sinusitis were defined according to the Center Disease Control and Prevention (CDC) criteria. The observation period was until extubation or up to a maximum of ten days after intubation, whatever occurred first. Successful extubation was defined as discharged alive from ICU without prior requirement for tracheostomy.

Weaning from the ventilator was guided by standard operating procedure. All patients received a spontaneous breathing trial daily after weaning readiness was reached, followed by predefined weaning steps corresponding to predefined lengths of spontaneous breathing. A RASS of − 1 was aimed for with the least possible dose of sedatives, but still ensuring adequate tolerance to the endotracheal tube. RASS was obtained by the nursing staff at least three times daily and documented in the electronic record.

The ICU mobility scale includes scores from 0 (passive exercise by staff, patient not actively moving) to 10 (walking independently without a gait aid) with (1) exercises in bed, (2) patient passively moved to chair, (3) sitting over edge of bed, (4) standing, (5) transfer bed to chair, (6) marching on spot at bedside, and scores above (7) walking away from bed.

The length of ventilation, rates of successful extubation and tracheostomy were obtained from the last instance of intubation.

### Data retrieval

Data were obtained anonymized from the patient data management system (Intensive Care Manager V10 and the associated data extraction tool ICMiq V1.3, both Drägerwerk AG, Lübeck, Germany) in the Department of Intensive Care Medicine (University Medical Center Hamburg-Eppendorf, Germany). Data were managed with Microsoft Excel 365 and Visual Basic V7.1 (both Microsoft Inc., Redmond, WA, USA).

### Statistics

Statistical analyses were performed using SPSS (version 27, IBM Inc., Armonk, NY, USA). We used t-tests, Welsh tests, Fisher’s Exact tests, binary multivariable logistic regression analyses, and generalized mixed model analyses, as applicable. For the logistic regression analyses, parameters differing with a p-value < 0.1 between the groups were tested as covariates. Furthermore, we conducted sensitivity analyses included only patients with one instance of intubation, excluding patients’ diagnoses, including COVID-19 disease as a separate group, and excluding patients suffering from COVID-19 from the analysis.

## Results

**Figure 1:**
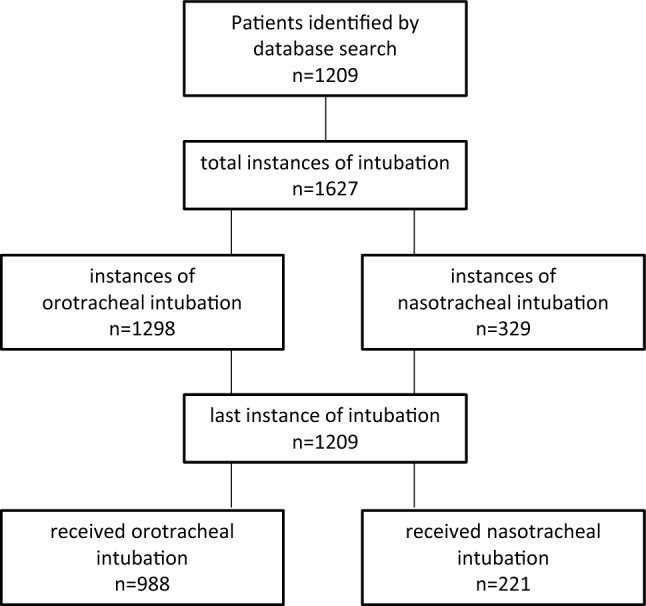
Patient inclusion and outcome. OTI: orotracheal intubation group, NTI: nasotracheal intubation group.

From January 1, 2018 to December 31, 2020, 1209 patients were identified from the electronic database with 1627 instances of tracheal intubation and ventilation for more than 48 h. An overview on patients’ characteristics and disease categories is given in Table 1. In the category of community acquired pneumonia, 43 Patients in the OTI versus 0 patients in the NTI group suffered from COVID-19, p < 0.001. Patient inclusion is depicted in Fig. 1 and outcome, and length of ventilation are shown in Table 2. SOFA scores decreased in both groups over the observation period (Supplementary Fig. S1).

**Table 1:** Patients’ characteristics.

Parameter	Orotracheal intubation (n = 988)	Nasotracheal intubation (n = 221)	p-value
Age (years)	63 ± 15	66 ± 13	0.002
Weight (kg)	78.8 ± 18.6	79.5 ± 21.5	0.615
Height (cm)	173 ± 10	173 ± 10	0.461
BMI (kg/m^2^)	26.4 ± 6.8	27.2 ± 12.5	0.236
Length of ICU stay (d)	26 ± 26	26 ± 28	0.704
Length of ventilation (d)	6.9 ± 4.8	5.8 ± 4.0	< 0.001
APACHE II-Score	31 ± 7	31 ± 8	0.111
SAPS II	55 ± 18	54 ± 18	0.359
Initial SOFA-Score	11 ± 3	10 ± 3	< 0.001
Patients’ disease category			
Medical	374 (38%)	69 (31%)	0.076
Community-acquired pneumonia	124 (13%)	22 (10%)	0.306
Neurology	178 (18%)	63 (29%)	0.001
Surgical	312 (32%)	67 (30%)	0.749

BMI: Body mass index, ICU: intensive care unit, APACHE: Acute Physiology And Chronic Health Evaluation, SAPS: Simplified Acute Physiology Score, SOFA: sequential organ failure assessment.

**Table 2:** Patients’ outcomes.

Parameter	Orotracheal intubation (n = 988)	Nasotracheal intubation (n = 221)	p-value
Extubated (n)	335 (34%)	83 (38%)	0.141
Tracheostomy (n)	232 (23%)	75 (34%)	0.141
Deceased (n)	421 (43%)	63 (28%)	< 0.001
Length of ventilation (d)			
Before extubation	5.6 ± 3.7	5.2 ± 4.3	0.408
Before tracheostomy	7.3 ± 4.9	5.7 ± 3.2	0.001
Before death	7.3 ± 5.1	6.3 ± 3.5	0.113

**Figure 2:**
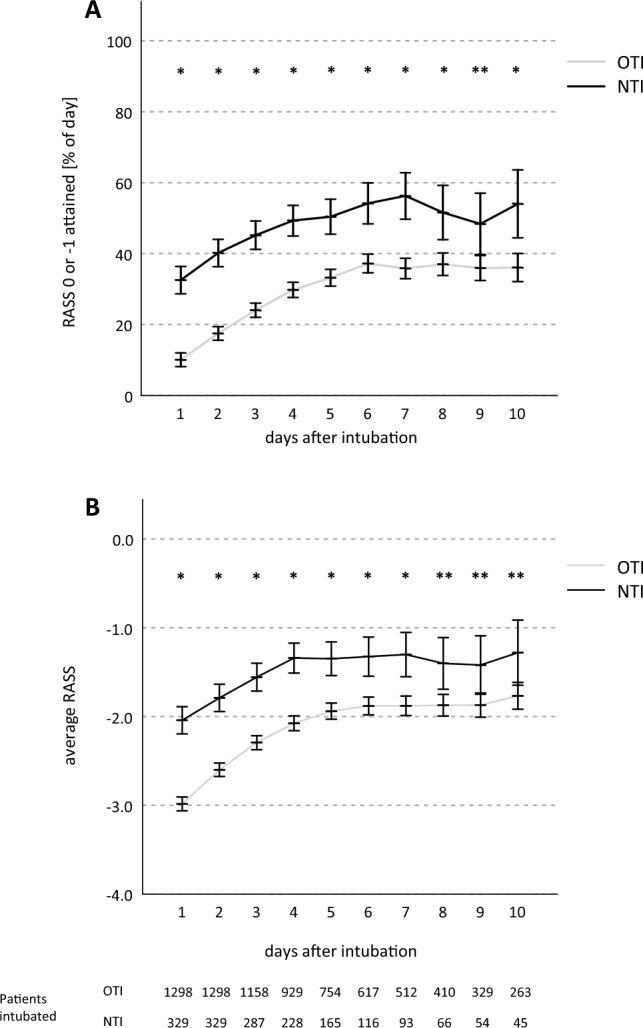
Richmond Agitation Sedation Scale. RASS: Richmond Agitation Sedation Scale, OTI: orotracheal intubation group, NTI: nasotracheal intubation group, *p < 0.001, **p < 0.05.

From day 1 to the end of day 3, a RASS score of 0 or − 1 was observed for 4.0 ± 6.1 h/d in the OTI group versus 9.4 ± 8.4 h/d in patients in the NTI group, p < 0.001. For the complete observation period to the end of day 10, patients attained the targeted scores for 5.7 ± 6.2 h/d in the OTI group versus 10.7 ± 8.0 h/d in the NTI group, p < 0.001 (Fig. 2A). On day 1, mean RASS values were − 3.0 ± 0.0 in the OTI group versus − 2.0 ± 0.1 in the NTI group (p < 0.001), increasing in both groups and reaching a plateau in the OTI group on day 5 at − 2.0 ± 0.0 and on day 4 at − 1.3 ± 0.1 in the NTI group. In the mixed model analysis, mean RASS was significantly different between the groups (d1–d7: p < 0.001, d8: p = 0.003, d9: p = 0.013, d10: p = 0.016) with higher values in the NTI group (Fig. 2B).

Patients in the NTI group received propofol, sufentanil, and norepinephrine less frequently than patients in the OTI group (supplementary table S1). Furthermore, NTI patients received lower doses of propofol, sufentanil, and norepinephrine. Detailed information on doses is given in the supplement, Figs. S2–S4 and Tables S2–S4.

**Figure 3:**
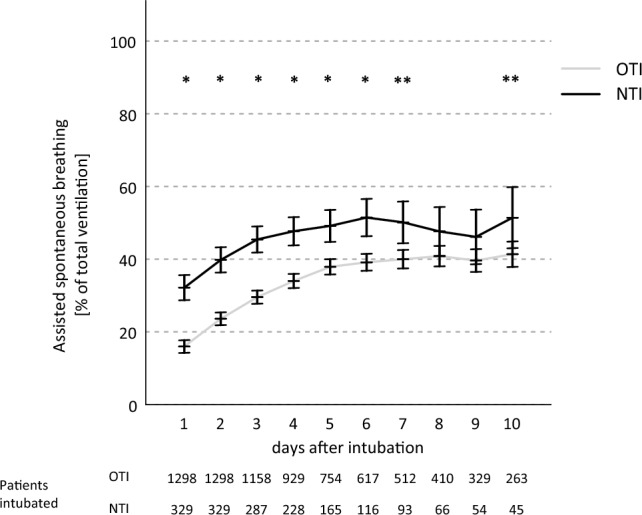
Proportion of assisted spontaneous breathing. OTI: orotracheal intubation group, NTI: nasotracheal intubation group, *p < 0.001, **p < 0.05.

**Figure 4:**
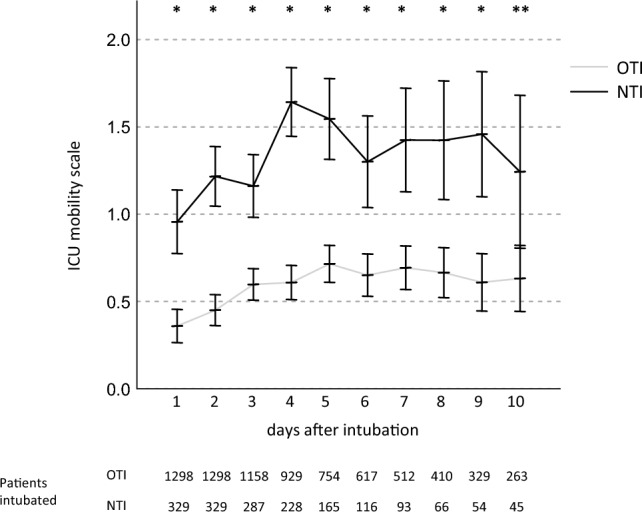
Mobilization by physiotherapy. ICU: intensive care unit, OTI: orotracheal intubation group, NTI: nasotracheal intubation group, *p < 0.001, **p < 0.05.

Of the total time of mechanical ventilation, patients in the NTI group showed a higher proportion of spontaneous breathing (Fig. 3) from day 1 to 7 (day 1–6: p < 0.001, day 7: p = 0.002). Over the observation period, ICU-MS scores were consistently higher in the NTI group (d1 to d9: p < 0.001, d10: p = 0.012), see Fig. 4. VAP occurred in 19.1% of instances of ventilation in the OTI group and 19.9% in the NTI group, p = 0.728. Clinically apparent sinusitis occurred in 0.2% in the OTI group versus 0.3% in the NTI group, p = 0.807.

Four hundred eighty-four patients (40%) died during the intensive care stay. OTI, age, initial SOFA scores, length of ventilation, and the number of intubations were independent predictors for mortality while mortality was lower in neurological and surgical patients (Table 3). The sensitivity analyses including only patients with one instance of intubation, excluding patients’ diagnoses, including patients with COVID-19 disease as a separate group, or excluding COVID-19 patients from the analysis yielded similar results (supplementary tables S5).

**Table 3:** Independent predictors for mortality.

Parameter	Odds ratio	95% confidence intervals		p-value
OTI	1.602	1.132	2.268	0.008
Age (y)	1.029	1.020	1.039	< 0.001
SOFA on day of intubation	1.181	1.131	1.233	< 0.001
Length of ventilation (d)	1.037	1.009	1.066	0.009
number of intubations (n)	0.886	0.788	0.997	0.044
Patients’ disease category:				
Medical	1	Reference		
Community-acquired pneumonia	0.866	0.573	1.308	0.494
Neurology	0.289	0.194	0.429	< 0.001
Surgical	0.549	0.405	0.745	< 0.001

Multivariable logistic regression analysis. OTI: orotracheal intubation group, SOFA: Sequential Organ Failure Assessment, number of intubations: cumulative number of intubations with subsequent mechanical ventilation longer than 48 h.

## Discussion

In this retrospective study, we evaluated the effects of nasotracheal versus orotracheal intubation in critically ill patients ventilated for at least 48 h. Patients who received nasotracheal intubation were more alert, required lower doses of sedative drugs as well as vasopressors, and could be mobilized to a higher degree during physiotherapy. Furthermore, our data showed an independent association between orotracheal intubation and mortality, even after adjustment for disease severity by SOFA scores and duration of ventilation.

Light versus deep sedation has been shown to decrease mortality and to reduce the duration of mechanical ventilation^3,12,13^. A RASS target of 0 or − 1 has been recommended by guidelines for intensive care patients^14^. Patients who received nasotracheal intubation required less sedative medication and therefore spent more time in the favorable RASS range. We suggest that the reason for less sedation is the reduced oropharyngeal stimulus causing pharyngeal reflexes that need to be attenuated in order to tolerate an orally placed airway device. Two recent studies showed that tracheostomy decreases the doses of required sedative drugs and significantly increases RASS values as compared to before intervention^15,16^. In patients with tracheostomy, no stimulus exists that induces pharyngeal reflexes leading to gagging^17^. Therefore, no or less sedation is required in order to tolerate a tracheal cannula placed via tracheostomy. In patients with a nasopharyngeal or nasotracheal airway, the tube only causes slight triggering of pharyngeal reflexes, presumably explaining the requirement of less sedative medication.

Patients in the NTI group also required less vasopressor therapy and showed a higher proportion of spontaneous breathing. We deem this to be linked to the requirement of less sedative medication: in the majority of patients, propofol was used which is a potent vasodilator^18^. This explains the higher doses of norepinephrine in the OTI group to maintain a sufficient mean arterial pressure. Furthermore, opioids cause respiratory depression and sufentanil requirements were higher in the OTI group causing less spontaneous breathing in this group. Less sedation with patients being more alert could also explain why patients in the NTI group could be mobilized to a higher degree during physiotherapy.

More patients died in the OTI group as compared to the NTI group. Patients in the OTI group had higher SOFA scores which have been shown to predict mortality in various patient cohorts although initially the score had been designed as an indicator of organ dysfunction and not for outcome^19–21^. Interestingly, APACHE II scores and SAPS II designed to predict mortality did not differ between our groups. Predicted mortality according to SAPS II and the initial SOFA score was 55% and 40–50% respectively, which is congruent to the observed mortality of 40% in our cohort. The higher mortality in the OTI group may be in part attributable to the higher degree of organ dysfunction as indicated by the higher SOFA scores. However, OTI could also be identified as an independent predictor for mortality, taking SOFA scores into account as a covariate. One explanation could be that OTI patients required more sedation which in itself may be harmful and several studies have shown an association between deep sedation and a decrease of survival^2,3,22^.

The length of ventilation was similar in both groups prior to extubation. However, patients in the NTI group received tracheostomy earlier than the OTI group. The optimum timing of tracheostomy in critically ill patients is still not well defined. A meta-analysis found that early tracheostomy was associated with a reduced incidence of VAP and ventilator free days, and a reduced mortality^23^, while the effect on mortality was comparable between early (before day 7) and late tracheostomy in a more recent meta-analysis^24^. Considering only patients with acute brain injury, early tracheostomy defined as before or at day 10 after intubation was associated with a reduced long-term mortality^25^ while a recent trial in stroke patients did not find a difference in outcome^26^. Most patients in our cohort received an early tracheostomy as per definition of the meta-analyses. In our cohort, we suggest that lower doses of sedative medication allowed for an earlier neurological and functional assessment of swallowing and cough reflexes and thus the possibility of extubation versus the requirement for tracheostomy in the NTI group. Therefore, a decision on further treatment could be made earlier explaining the shorter time of ventilation before tracheostomy.

The reported incidence of sinusitis associated with nasotracheal intubation varies from 2.3% to above 40%^6,9,27,28^. In a study by Pedersen et al., only one sinusitis occurred in 357 patients ventilated for less than five days, but the incidence of sinusitis increased to 49% after day five, although this sharp rise in the incidence may be attributable to the study design mandating sinus X-ray after day five^28^. In our study cohort, only one sinusitis was clinically apparent in 326 instances of nasotracheal intubation, but our mean duration of ventilation was below six days in the NTI group. However, sinusitis may easily be overlooked: using a systematic approach, Holzapfel et al. found signs of sinusitis in 40% of patients who were then treated accordingly while no sinusitis was apparent in the control group without a systematic search for signs of sinusitis^29^. Interestingly, the incidence of VAP and mortality were higher in the control group, as well, while an increase in mortality was primarily attributed to the observed increase of pneumonia. However, in our cohort, no protocol for the systematic search for sinusitis existed and incidence of VAP was similar in both groups. Van Zandten et al. could show that sinusitis is a typical cause of fever of unknown origin in critically ill patients^8^. The mechanism for sinusitis in nasotracheal intubation may be the occlusion of the maxillary sinus ostium in the middle nasal meatus and it has been shown that tracheal tubes are placed there in 83% of cases^30^. However, tubes used in that study were predominantly Ring–Adair–Elwyn (RAE) tubes and it is unclear if the higher stiffness compared to the Woodbrigde tubes regularly used in our hospital for nasotracheal intubation promotes positioning of the tube in the middle instead of the lower nasal meatus. No systematic data exists on the typical positioning of Woodbridge tubes in nasotracheal intubation, so far.

Our study has certain limitations. We analyzed data from a single center which limits generalizability to other patient populations, e.g. due to different sedation protocols or different treatment practices. We analyzed retrospective data and therefore can only show associations without causality. However, we conducted an exploratory analysis to generate hypothesis for forthcoming prospective trials. The choice of airway route may not have been completely random, and there were no predefined criteria as to which patients should receive NTI or OTI. Due to our retrospective approach, we cannot exclude that the higher observed mortality in the OTI group is attributable to unidentified confounders and insufficient adjustment of our logistic regression analysis. We did not systematically look for sinusitis as a potential complication and may have had a far higher incidence in the NTI group that remained clinically inapparent. Unfortunately, we are not able to report the reason for ICU admission, and the precise diagnosis because these data were not retrievable from our electronic records. Finally, the maximum observation period of ten days after intubation was chosen arbitrarily.

## Conclusion

In our retrospective analysis of nasotracheal versus orotracheal intubation in critically ill patients, we could show that nasotracheal intubation was associated with fewer requirements of sedative medication, more assisted spontaneous breathing, and a higher degree of mobilization during physiotherapy. The incidences of clinically apparent sinusitis and VAP were similar in both groups. Interestingly, the orotracheal intubation route was an independent risk factor for mortality. Due to our data, we deem a new prospective evaluation of nasotracheal versus orotracheal intubation necessary to study risks and benefits in current critical care medicine.

### Supplementary Information

Supplementary Information.

## Data Availability

The data are available from the corresponding author upon reasonable request.

## Abbreviations

APACHE: Acute physiology and chronic health evaluation
ICU-MS: Intensive care unit mobility scale
NTI: Nasotracheal intubation
OTI: Orotracheal intubation
RAE: Ring–Adair–Elwyn
RASS: Richmond agitation sedation scale
SAPS: Simplified acute physiology score
SOFA: Sequential organ failure assessment
VAP: Ventilator associated pneumonia
