# Supplementary material for: Comparison of nasotracheal versus orotracheal intubation for sedation, assisted spontaneous breathing, mobilization, and outcome in critically ill patients: an exploratory retrospective analysis
Source: Sci Rep. 2023 Aug 3;13:12616. doi: 10.1038/s41598-023-39768-1 (PMC10400581; doi:10.1038/s41598-023-39768-1)
Supplement: Supplementary file 1 — Supplementary Information. [file 41598_2023_39768_MOESM1_ESM.docx]

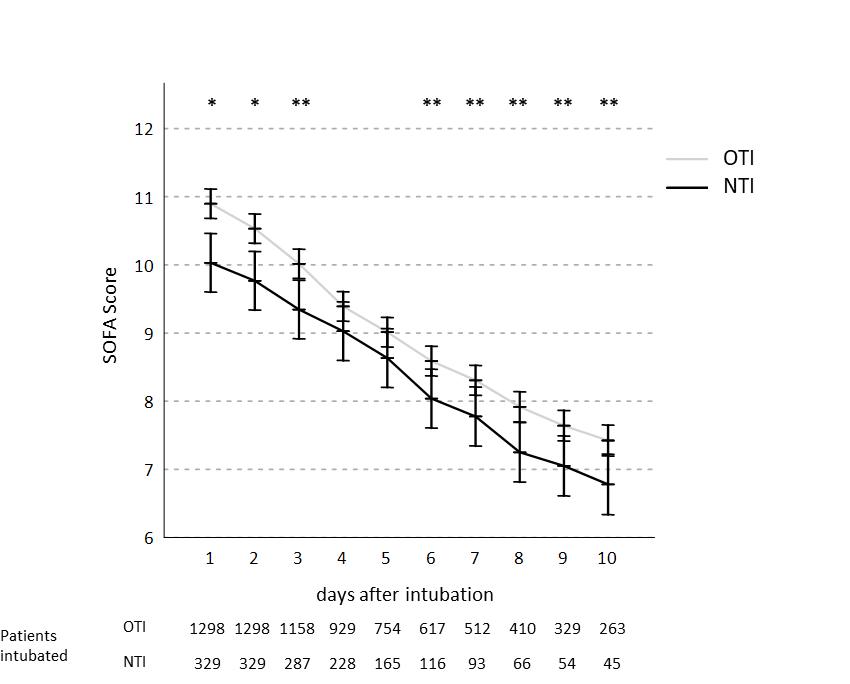


Figure S1: SOFA scores

SOFA: sequential organ failure assessment, OTI: orotracheal intubation, NTI: nasotracheal intubation, * p<0.001, ** p<0.05.

Table S1: Use of medication

| **Drug** | **OTI (n=1298)** | **NTI (n=329)** | **p** |
| --- | --- | --- | --- |
| Propofol | 1156 (89%) | 200 (61%) | <0.001 |
| Midazolam | 95 (7%) | 20 (6%) | 0.433 |
| Barbiturates | 9 (<1%) | 2 (<1%) | 0.866 |
| Ketamine | 28 (2%) | 3 (1%) | 0.140 |
| Sufentanil | 1196 (92%) | 218 (66%) | <0.001 |
| Norepinephrine | 1262 (97%) | 287 (87%) | <0.001 |

OTI: orotracheal intubation, NTI: nasotracheal intubation; barbiturates include thiopentone and methohexital.


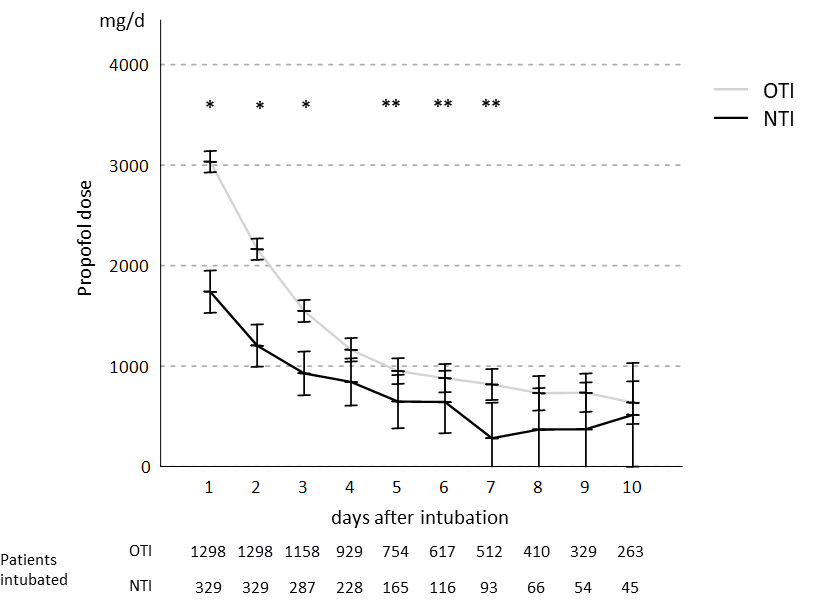


Figure S2: Propofol dose

OTI: orotracheal intubation, NTI: nasotracheal intubation, * p<0.001, ** p<0.05.

Table S2: Propofol dose

| Propofol dose | |  |  |  |  |  |  |  |
| --- | --- | --- | --- | --- | --- | --- | --- | --- |
| day | group | mg/d | 95% CI of mean | | difference | 95% CI of difference | | p |
| 1 | OTI | 3034 | 2928 | 3140 | 1294 | 1058 | 1529 | <0.001 |
|  | NTI | 1740 | 1530 | 1950 |  |  |  |  |
| 2 | OTI | 2163 | 2057 | 2269 | 960 | 724 | 1195 | <0.001 |
|  | NTI | 1204 | 993 | 1414 |  |  |  |  |
| 3 | OTI | 1548 | 1439 | 1657 | 621 | 378 | 864 | <0.001 |
|  | NTI | 927 | 710 | 1144 |  |  |  |  |
| 4 | OTI | 1161 | 1045 | 1278 | 320 | 58 | 582 | 0.017 |
|  | NTI | 842 | 607 | 1076 |  |  |  |  |
| 5 | OTI | 950 | 822 | 1078 | 304 | 9 | 599 | 0.044 |
|  | NTI | 646 | 380 | 912 |  |  |  |  |
| 6 | OTI | 880 | 739 | 1020 | 237 | -104 | 578 | 0.172 |
|  | NTI | 642 | 332 | 953 |  |  |  |  |
| 7 | OTI | 817 | 662 | 971 | 535 | 150 | 921 | 0.007 |
|  | NTI | 281 | -72 | 635 |  |  |  |  |
| 8 | OTI | 730 | 559 | 901 | 362 | -84 | 808 | 0.112 |
|  | NTI | 368 | -44 | 780 |  |  |  |  |
| 9 | OTI | 734 | 544 | 925 | 363 | -140 | 866 | 0.157 |
|  | NTI | 371 | -94 | 837 |  |  |  |  |
| 10 | OTI | 636 | 423 | 849 | 122 | -437 | 681 | 0.669 |
|  | NTI | 514 | -3 | 1031 |  |  |  |  |

OTI: orotracheal intubation, NTI: nasotracheal intubation, CI: confidence interval.


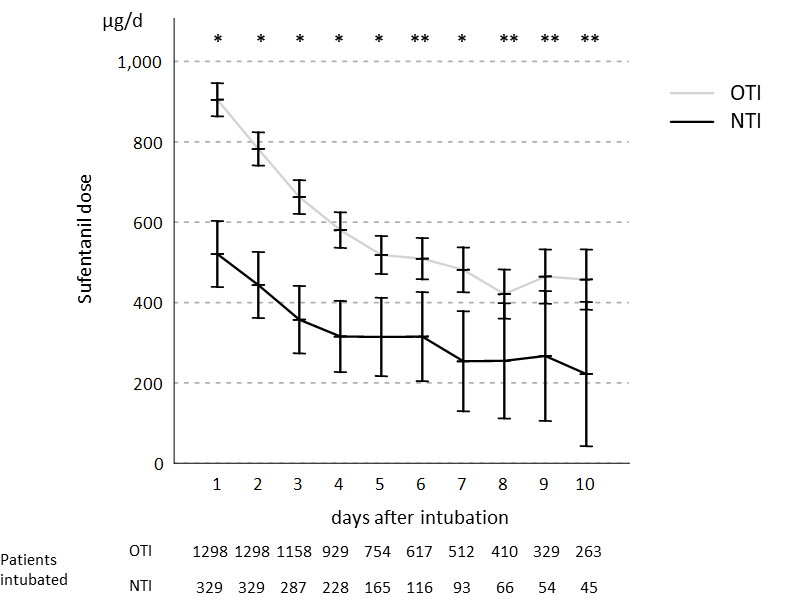


Figure S3: Sufentanil dose

OTI: orotracheal intubation, NTI: nasotracheal intubation, * p<0.001, ** p<0.05.

Table S3: Sufentanil dose

| Sufentanil dose | |  |  |  |  |  |  |  |
| --- | --- | --- | --- | --- | --- | --- | --- | --- |
| day | group | µg/d | 95% CI of mean | | difference | 95% CI of difference | | p |
| 1 | OTI | 905 | 863 | 946 | 384 | 292 | 476 | <0.001 |
|  | NTI | 521 | 439 | 603 |  |  |  |  |
| 2 | OTI | 782 | 741 | 824 | 339 | 245 | 430 | <0.001 |
|  | NTI | 444 | 362 | 526 |  |  |  |  |
| 3 | OTI | 663 | 621 | 705 | 305 | 211 | 399 | <0.001 |
|  | NTI | 358 | 274 | 441 |  |  |  |  |
| 4 | OTI | 580 | 536 | 625 | 265 | 166 | 364 | <0.001 |
|  | NTI | 315 | 227 | 404 |  |  |  |  |
| 5 | OTI | 519 | 471 | 566 | 204 | 96 | 312 | <0.001 |
|  | NTI | 315 | 217 | 412 |  |  |  |  |
| 6 | OTI | 509 | 458 | 561 | 194 | 72 | 316 | 0.002 |
|  | NTI | 315 | 205 | 426 |  |  |  |  |
| 7 | OTI | 482 | 426 | 537 | 227 | 91 | 364 | 0.001 |
|  | NTI | 254 | 130 | 379 |  |  |  |  |
| 8 | OTI | 421 | 360 | 482 | 166 | 10 | 322 | 0.037 |
|  | NTI | 255 | 112 | 399 |  |  |  |  |
| 9 | OTI | 465 | 397 | 532 | 198 | 23 | 373 | 0.027 |
|  | NTI | 267 | 106 | 429 |  |  |  |  |
| 10 | OTI | 457 | 382 | 532 | 235 | 41 | 430 | 0.018 |
|  | NTI | 222 | 43 | 402 |  |  |  |  |

OTI: orotracheal intubation, NTI: nasotracheal intubation, CI: confidence interval.


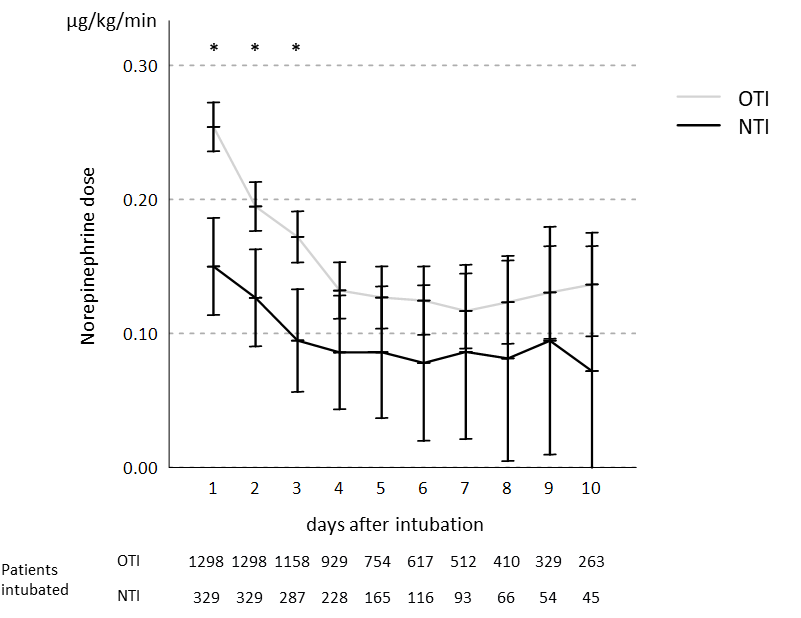


Figure S4: Norepinephrine dose

OTI: orotracheal intubation, NTI: nasotracheal intubation, * p<0.001.

Table S4: Norepinephrine dose

| Norepinephrine dose | | | | | | | | |
| --- | --- | --- | --- | --- | --- | --- | --- | --- |
| day | group | µg/kg/min | 95% CI of mean | | difference | 95% CI of difference | | p |
| 1 | OTI | 0.254 | 0.236 | 0.272 | 0.104 | 0.064 | 0.145 | <0.001 |
|  | NTI | 0.150 | 0.114 | 0.186 |  |  |  |  |
| 2 | OTI | 0.195 | 0.176 | 0.213 | 0.068 | 0.028 | 0.109 | <0.001 |
|  | NTI | 0.127 | 0.090 | 0.163 |  |  |  |  |
| 3 | OTI | 0.172 | 0.153 | 0.191 | 0.077 | 0.034 | 0.120 | <0.001 |
|  | NTI | 0.095 | 0.056 | 0.133 |  |  |  |  |
| 4 | OTI | 0.132 | 0.111 | 0.153 | 0.046 | -0.001 | 0.094 | 0.056 |
|  | NTI | 0.086 | 0.043 | 0.128 |  |  |  |  |
| 5 | OTI | 0.127 | 0.104 | 0.150 | 0.041 | -0.014 | 0.095 | 0.141 |
|  | NTI | 0.086 | 0.037 | 0.135 |  |  |  |  |
| 6 | OTI | 0.125 | 0.099 | 0.150 | 0.047. | -0.017 | 0.110 | 0.150 |
|  | NTI | 0.078 | 0.020 | 0.136 |  |  |  |  |
| 7 | OTI | 0.117 | 0.089 | 0.145 | 0.031 | -0.040 | 0.101 | 0.397 |
|  | NTI | 0.086 | 0.021 | 0.151 |  |  |  |  |
| 8 | OTI | 0.123 | 0.092 | 0.154 | 0.042 | -0.041 | 0.125 | 0.319 |
|  | NTI | 0.081 | 0.005 | 0.158 |  |  |  |  |
| 9 | OTI | 0.131 | 0.096 | 0.165 | 0.036 | -0.056 | 0.128 | 0.442 |
|  | NTI | 0.095 | 0.010 | 0.180 |  |  |  |  |
| 10 | OTI | 0.137 | 0.098 | 0.175 | 0.065 | -0.036 | 0.166 | 0.210 |
|  | NTI | 0.072 | -0.021 | 0.165 |  |  |  |  |

OTI: orotracheal intubation, NTI: nasotracheal intubation, CI: confidence interval.

Table S5: Sensitivity analyses – logistic regression analysis (mortality)

## A) Patients with only one instance of intubation

|  | Orotracheal intubation  (n = 719) | Nasotracheal intubation  (n = 135) | p-value |
| --- | --- | --- | --- |
| mortality | 334 (46 %) | 45 (33 %) | 0.007 |

Independent predictors for mortality

| **Parameter** | **Odds ratio** | **95% confidence intervals** | | **p-value** |
| --- | --- | --- | --- | --- |
| OTI | 1.612 | 1.072 | 2.424 | 0.022 |
| Age [y] | 1.023 | 1.012 | 1.033 | <0.001 |
| SOFA on day of intubation | 1.191 | 1.137 | 1.247 | <0.001 |
| Length of ventilation [d] | 1.036 | 1.006 | 1.067 | 0.017 |

Multivariable logistic regression analysis. OTI: orotracheal intubation group, SOFA: Sequential Organ Failure Assessment.

## B) Patients’ diagnoses excluded in logistic regression analysis

Independent predictors for mortality

| **Parameter** | **Odds ratio** | **95% confidence intervals** | | **p-value** |
| --- | --- | --- | --- | --- |
| OTI | 1.654 | 1.178 | 2.322 | 0.004 |
| Age [y] | 1.026 | 1.017 | 1.035 | <0.001 |
| SOFA on day of intubation | 1.211 | 1.161 | 1.262 | <0.001 |
| Length of ventilation [d] | 1.039 | 1.012 | 1.067 | 0.004 |
| number of intubations [n] | 0.843 | 0.751 | 0.947 | 0.004 |

Multivariable logistic regression analysis. OTI: orotracheal intubation group, SOFA: Sequential Organ Failure Assessment.

## C) COVID-19 disease as separate group

Independent predictors for mortality

| **Parameter** | **Odds ratio** | **95% confidence intervals** | | **p-value** |
| --- | --- | --- | --- | --- |
| OTI | 1.584 | 1.118 | 2.244 | 0.010 |
| Age [y] | 1.029 | 1.020 | 1.039 | <0.001 |
| SOFA on day of intubation | 1.182 | 1.132 | 1.234 | <0.001 |
| Length of ventilation [d] | 1.036 | 1.008 | 1.065 | 0.010 |
| number of intubations [n] | 0.887 | 0.789 | 0.997 | 0.044 |
| **Patients’ disease category:** |  |  |  |  |
| Medical | 1 | Reference | | |
| COVID-19 | 1.064 | 0.537 | 2.110 | 0.858 |
| Community-acquired pneumonia | 0.795 | 0.497 | 1.273 | 0.304 |
| Neurology | 0.289 | 0.195 | 0.430 | <0.001 |
| Surgical | 0.549 | 0.405 | 0.745 | <0.001 |

Multivariable logistic regression analysis. OTI: orotracheal intubation group, SOFA: Sequential Organ Failure Assessment.

## D) Patients’ with COVID-19 excluded in logistic regression analysis

Independent predictors for mortality

| **Parameter** | **Odds ratio** | **95% confidence intervals** | | **p-value** |
| --- | --- | --- | --- | --- |
| OTI | 1.596 | 1.126 | 2.263 | 0.009 |
| Age [y] | 1.030 | 1.020 | 1.040 | <0.001 |
| SOFA on day of intubation | 1.184 | 1.133 | 1.238 | <0.001 |
| Length of ventilation [d] | 1.031 | 1.002 | 1.061 | 0.036 |
| number of intubations [n] | 0.890 | 0.792 | 1.001 | 0.052 |
| **Patients’ disease category:** |  |  |  |  |
| Medical | 1 | Reference | | |
| Community-acquired pneumonia | 0.807 | 0.504 | 1.292 | 0.372 |
| Neurology | 0.290 | 0.195 | 0.431 | <0.001 |
| Surgical | 0.546 | 0.402 | 0.740 | <0.001 |

Multivariable logistic regression analysis. OTI: orotracheal intubation group, SOFA: Sequential Organ Failure Assessment.

Table S6: RASS 0 or -1 from day 1 to 3 according to disease categories

| Disease category | Orotracheal intubation  (n = 1298) | Nasotracheal intubation  (n = 329) | p-value |
| --- | --- | --- | --- |
| Medical | 3.3 ± 5.9 h/d | 10.1 ± 9.0 h/d | <0.001 |
| Community-acquired pneumonia | 2.8 ± 5.4 h/d | 6.1 ± 8.2 h/d | 0.031 |
| Neurology | 6.3 ± 7.4 h/d | 10.8 ± 8.9 h/d | <0.001 |
| Surgical | 4.0 ± 5.6 h/d | 9.6 ± 8.0 h/d | <0.001 |

Data are given as mean ± standard deviation. All instances of intubation included.

Table S7: Reason for intubation

| Reason | Orotracheal intubation  (n = 1298) | Nasotracheal intubation  (n = 329) | Total (n=1627) |
| --- | --- | --- | --- |
| Airway* | 81 | 24 | 105 |
| Cardiogenic shock/ heart failure | 62 | 27 | 89 |
| Cardio-pulmonary resuscitation | 44 | 6 | 50 |
| Decreased level of consciousness | 318 | 67 | 385 |
| Hemorrhagic shock | 5 | 6 | 11 |
| Respiratory or ventilatory insufficiency | 606 | 187 | 793 |
| Septic shock | 182 | 12 | 194 |

*includes insufficient airway patency and airway obstruction e.g. to bleeding
